# Three-dimensional visualization of cultural clusters in the 1878 yellow fever epidemic of New Orleans

**DOI:** 10.1186/1476-072X-7-47

**Published:** 2008-08-22

**Authors:** Andrew J Curtis

**Affiliations:** 1 GIS Research Laboratory, Department of Geography, University of Southern California, Kaprielian Hall (KAP), Room 416, 3620 South Vermont Avenue, Los Angeles, CA 90089-0255, USA

## Abstract

**Background:**

An epidemic may exhibit different spatial patterns with a change in geographic scale, with each scale having different conduits and impediments to disease spread. Mapping disease at each of these scales often reveals different cluster patterns. This paper will consider this change of geographic scale in an analysis of yellow fever deaths for New Orleans in 1878. Global clustering for the whole city, will be followed by a focus on the French Quarter, then clusters of that area, and finally street-level patterns of a single cluster. The three-dimensional visualization capabilities of a GIS will be used as part of a cluster creation process that incorporates physical buildings in calculating mortality-to-mortality distance. Including nativity of the deceased will also capture cultural connection.

**Results:**

Twenty-two yellow fever clusters were identified for the French Quarter. These generally mirror the results of other global cluster and density surfaces created for the entire epidemic in New Orleans. However, the addition of building-distance, and disease specific time frame between deaths reveal that disease spread contains a cultural component. Same nativity mortality clusters emerge in a similar time frame irrespective of proximity. Italian nativity mortalities were far more densely grouped than any of the other cohorts. A final examination of mortalities for one of the nativity clusters reveals that further sub-division is present, and that this pattern would only be revealed at this scale (street level) of investigation.

**Conclusion:**

Disease spread in an epidemic is complex resulting from a combination of geographic distance, geographic distance with specific connection to the built environment, disease-specific time frame between deaths, impediments such as herd immunity, and social or cultural connection. This research has shown that the importance of cultural connection may be more important than simple proximity, which in turn might mean traditional quarantine measures should be re-evaluated.

## Background

The spatial pattern of disease cases in an epidemic, and the apparent process of diffusion, will vary dependent on the geographic scale of investigation. Although it is commonly accepted that such geographic variation exists, a lack of suitable epidemic data at the individual case level has proved limiting in visualizing these scale variations. This paper uses mortality data from the yellow fever epidemic of 1878 in New Orleans, with a focus on the French Quarter, to consider both variations in scale-dependent disease pattern, and the impact the built environment and culture plays in the formation of those patterns. The ability to investigate these last two aspects of the epidemic, both of which have previously been identified as important in understanding the nuances of disease spread [1], is made possible by combining information about disease cases and contemporary building information within a three-dimensional GIS visualization.

### Yellow fever and New Orleans

Yellow fever is a virus of the genus *Flavivirus*, family Togaviridae, its symptomology often including haemorrhaging and jaundice from which the disease gained its name [2]. The major epidemics of New Orleans which ranged between 1853 and 1905 were vectored by *Aedes aegypti *mosquitoes [3]. This is a residential mosquito which prefers human blood, feeding most frequently during low light periods such as at dusk. It is unlikely the yellow fever virus "over wintered" in the city's mosquito population. Instead the large mosquito population became freshly infected each year by disease introduction from one of the steady stream of trade ships and/or immigrants entering the city. The temporal sequence of disease occurrence in New Orleans was a start around early to mid summer, the epidemic hitting full stride in August and September, before tailing out in November as the colder weather curtailed mosquito activity.

Although several yellow fever epidemics occurred in the southern United States during the 1800s, the most notable of which were in 1853 and 1878, a common pattern among them was an initial entry through a Gulf Coast city (most frequently New Orleans), followed by simultaneous diffusion patterns at different geographic scales [4,5]. By reading daily newspaper accounts of the period, and from Board of Health reports written following the outbreak [6,7], intra and inter urban diffusion patterns can be discerned. For example, in New Orleans two sailors were attributed to starting the 1878 epidemic by disembarking the *Emily B Souder*. This steamboat had just arrived from disease-impacted Cuba. The Board of Health reports went on to describe how personal connections set on a backcloth of the built environment, which included named streets, residences and shared courtyards, helped to spread the disease from its point of origin. Although it is unlikely these diffusion patterns occurred exactly as described, individual buildings are likely to have played an important role in disease spread. Indeed, Carter [8,9] used multiple empirical examples to prove this relationship. The importance of such close-proximity connections is now acknowledged as being important [10-12].

Once the initial disease introduction had occurred into New Orleans, the subsequent diffusion process would involve multiple scales, and locations, as the disease ran through the city, while simultaneously spreading along transportation routes, especially the Mississippi River [5] with both Vicksburg, Mississippi and Memphis, Tennessee being severely hit in 1878. The continuing diffusion of disease in New Orleans was now mirrored in other infected cities, which in turn began to infect neighbouring settlements. This complex diffusion made this the most *geographically *interesting epidemic in the 1800s.

The 1878 epidemic also signalled a shift in the vulnerability of the young to the contagion. The common perception of the time that yellow fever was a "strangers disease" resulted from the apparent immunity of families native to New Orleans [13]. The hardiness of those born in New Orleans resulted from childhood infection imparting life-long immunity. It appears that for most of the 1800s childhood infection was less serious and usually did not even result in a diagnosis of yellow fever. As a result this meant that each disease season meant little to the established families of the city who were more concerned about the potential economic losses incurred through quarantine than for the safety of their fellow citizens [4]. An analysis of the 1853 New Orleans epidemic found that a 20-year-old male immigrant was 11 to 37 times more likely to die than a 20-year-old native [14], and that the least vulnerable cohort were indeed children [15]. However, this trend was reversed in the 1878 epidemic, with children now becoming susceptible, in fact 58% of the total death toll were to children under the age of 16, with the mode age of death being 4 years [4,16,17]. This resulted in more widespread consternation as it soon became clear the disease was crossing socio-economic, ethnic and therefore geographic barriers in the city.

The 1878 epidemic is suitable for a multiple-scale investigation because of the spatial richness of the mortality data. The *Official Report of the Deaths from Yellow Fever as Reported by the New Orleans Board of Health *(ORD from this point on), written in the following year, detailed the mortality role of the epidemic, listing the name, age, nativity, place of residence and date of death [18]. Although other examples of historic medical sources have been the subject of spatial or GIS analysis [19,20], mortality data containing a spatial reference such as that found in the ORD, allow for more flexibility in GIS manipulation [21]. For this paper an initial analyses of mortality for the entire epidemic at the city-wide scale for New Orleans is followed by a focus on the French Quarter, then disease clusters found inside this area, and finally to residential patterns of these disease sequences.

A previous investigation of the ORD for the majority of New Orleans incorporated a spatial (50 m mosquito "flight range") and temporal sequence of disease deaths based on the extrinsic incubation period of the mosquito to identify a spatial variant of the basic reproduction number [17]. This approach, however, did not include subtleties of the built environment in the distance calculation, for example flight-line across shared inner courtyards. Nor did it consider the role of cultural diffusion. This is an important point as mathematical epidemiologists accept that spatial diffusion is likely to be impacted by cultural space and social networks [22-24], though these are known to be extremely difficult to model because of their complexity [1].

This paper, however, demonstrates that a GIS can be used to include both complex geography and cultural interconnectivity when mapping and analysing disease spread. Specifically this paper addresses two questions; are different spatial patterns of disease occurrence observable at different geographic scales of analysis? And can the incorporation of the built environment, and cultural connection, be used to identify disease clusters?

## Methods

Mortality locations were extracted from the ORD, and mapped in a GIS containing georegistered Sanborn and Robinson Fire Insurance maps which had been georegistered in ArcMap 9.1 (ESRI, Redlands, CA). This georegistration was achieved by adding known coordinate locations to sections of the French Quarter which have remained unchanged between 1878 and now. The detail in Sanborn Insurance maps (at a scale of one inch to 50 feet), is such that actual building addresses and other information relevant to the built environment, including number of floors, and the building's functional use can be identified. The 1878 city directory was used to verify that the address numbering scheme was the same for both the map creation year (1880 for Robinson, and 1885 for the Sanborn) and the year of the epidemic. As street names and address ranges were different to 2008 New Orleans, and therefore not compatible with available GIS layers, traditional address-matching approaches were impossible to perform. Other GIS based investigations of historic epidemics have created "new" street networks to allow for this approach [21]. For this paper each mortality was heads-up digitised into the correct building. Building footprints for the French Quarter were also heads-up digitised from the Sanborn Maps. By joining the personal information found in the ORD to the mortality point locations, and by identifying whether a building was a residence or commercial property, several further manipulations of the data were possible. These included using Arc Scene (ESRI, Redlands, CA) to render the French Quarter in three-dimensions using the number of floors as the vertical height. Deaths were also aggregated to buildings allowing for a choropleth representation at different geographies, including individual structures and city blocks. Mortalities were also queried and extracted by various subpopulations, including nativity, age, or date of death.

Once these geographic layers were created, a series of commonly used spatial analyses were performed to identify areas with high intensities of disease. At the global scale (the city of New Orleans), a spatial variant of the basic reproduction number had identified neighbourhood specific index cases and subsequent number of connected deaths to this first case [17]. For this paper a Kernel Density surface (KD) identified yellow fever mortality hotspots for all of New Orleans (with hospital deaths removed). By classifying these resulting interpolated distributions by standard deviations, one hotspot (greater than three standard deviations) was revealed in the area immediately to the north of Jackson Square. This hotspot is located in the middle of the French Quarter, and relatively close to the banks of the Mississippi.

**Figure 1:**
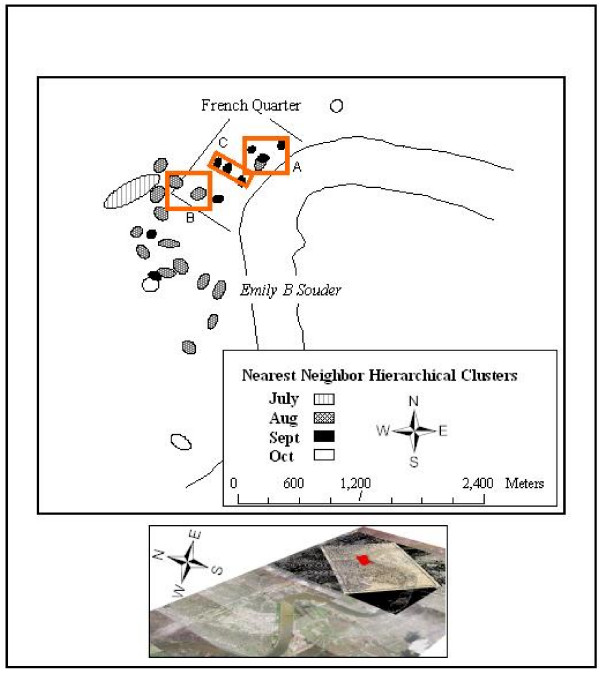
**Nearest Neighbor Hierarchical Clusters of Deaths For Each Month**. The probability for the clusters generated by nearest neighbor hierarchical clustering (NNHC) in Figure 1 is set at 0.05, the clusters displayed being first-order standard deviational ellipses. These ellipses reveal a similar pattern to the kernel density surface seen in Figure 2, namely, in the first part of the epidemic (up until August), statistically significant clusters follow a rough north-south line from the approximate area where the *Emily B Souder *docked, to the southeast of the French Quarter. Statistically significant ellipses also appear in the French Quarter for both August and especially September when the epidemic was at its peak (A to C in Figure 1). The inset map shows the location of the French Quarter on a yellow fever map of New Orleans from 1853, which in turn is overlaid on a current air photo of the larger city.

A nearest neighbour hierarchical clustering (NNHC) approach was also performed in Crimestat [25] to identify heavy mortality concentrations for each month of the epidemic (Figure 1). For this technique clusters are determined by the probability of distances between nearest neighbours occurring by chance (for an epidemiological example see [26]).

**Figure 2:**
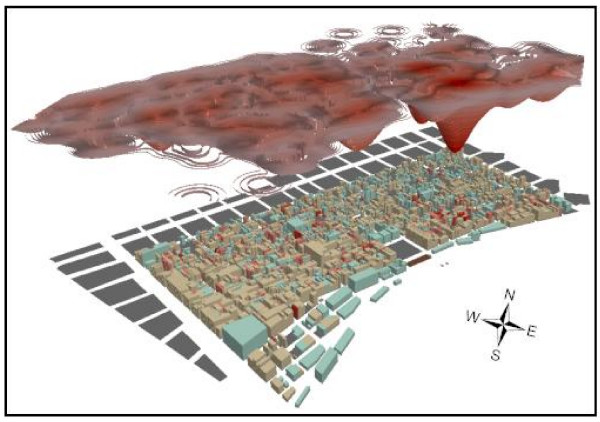
**A kernel density surface of yellow fever mortalities in the French Quarter**. The results of the kernel density surface created for all yellow fever mortalities falling in the French Quarter (n = 502) has been contoured, raised above the city and inversely manipulated by multiplying each contour by -0.1. This allows for the disease concentrations to be easily visualized with the three-dimensional buildings. The buildings in the city below are also coloured red (graded according to the number of mortalities each contains) and aqua if they are non-residential.

The results of these two preliminary investigations were used to focus the geographic setting of this paper. Apart from the French Quarter having a high number of deaths, it has the additional advantage of being one of the areas of New Orleans which maintains much of the same built environment today. This could allow for future investigations to actually visit the buildings and courtyards integral to disease spread. Also, the relatively equal residential density of the city blocks found in the French Quarter, though in no way a perfect surrogate for a population denominator, do offer a geographic way of controlling for unevenness in the underlying population surface. The lower population density in Uptown with its larger homes and generally higher socio-economic level would have posed problematic if used to compare mortality surfaces with the French Quarter.

A series of KD surfaces were generated for the French Quarter to investigate the spatial pattern of mortality at different spatial scales, and for different cultural subpopulations. In order to minimize boundary effects, a one block buffer including mortality locations around the French Quarter was included in the KD generation.

The first of these was a KD surface for all mortalities occurring in the French Quarter for the entire epidemic with no temporal consideration. This surface can still be revealing even without a denominator population in terms of showing where the greatest loss of life occurred.

This mortality surface was subdivided into four subpopulations: the three largest nativity populations (US, French and Italian born), and those under the age of 5. The rationale for the inclusion of the under 5 category was that this epidemic, as previously noted, had a severe toll among children. It was expected that a KD surface of children would present no clustering as, *ceteris paribus*, children were likely to be evenly distributed across all nativities. After a few years of residence, both immigrant and US born families would have US born children, this being reflected in a lack of spatial pattern for their mortalities.

For those assigned to the category of born in the United States, only ages greater than 10 were considered. This selection was made because children born to freshly landed immigrant families would otherwise confound patterns for "US born". Among the nativity locations included in this category were: Alabama, Baltimore, Boston, Chicago, Cincinnati, Illinois, Indiana, Iowa, Kentucky, Lafouche, Louisville, Louisiana, Maine, Maryland, Massachusetts, Michigan, Mississippi, New Jersey, New Orleans, New York, Ohio, Richmond, South Carolina, St Louis, Tennessee, Texas, and Virginia. For the French subgroup, Alsace was also included, and for the Italians, Sicily and Palermo were added as named nativity locations.

The KD surfaces involved no consideration of time, in other words the density surface only captures the overall pattern of death without including temporal subtleties, or phases, of the epidemic. The previously described calculation of the spatial basic reproduction number had included an appreciation of time sequences relevant to yellow fever, mainly taking into account viremia, days to death and the extrinsic incubation period of the mosquito [17]. However, the next analysis for this paper does account for temporal linkage while also investigating spatial characteristics of the built environment, and the impact of cultural distance.

Clusters of mortalities were created using the following criteria, each death was linked to a neighbouring death as long as each were within 50 meters, and both occurred within 21 days. As explained in Curtis et al (2007), 50 meters is an average flight distance of *Aedes aegypti*, though it was also suggested that the same distance could be used as a surrogate for cultural activity. This new clustering approach further focused on neighbour-to-neighbour connectivity by including that the distance had to be calculated by sight line, or along the same block face, within the three-dimensional built environment. Therefore, for two deaths to be considered as part of the same cluster, both had to be in buildings either on the same city block, visible from the building front to another building front (usually across the road), or from the back of the building across a shared courtyard. The temporal constraint of 21 days was selected as a conservative period linking deaths attributed to a shared index case which could result in both deaths occurring on the same day, or between 10 to 21 days if a mosquito feeds on the first infected individual, goes through extrinsic incubation, and then infects the second resulting in his/her death, a similar rationale to that employed in (Curtis et al 2007).

By linking mortalities in this manner, clusters were formed organically. In other words, as long as two deaths (A and B) could be linked in the manner described above, a third mortality need only be linked to A *or *B. As a result, clusters were expected to occupy different shapes based on the built environment, and not forming more classic circular patterns. As a result, these clusters are visualized as linked buildings, containing both those residences with mortalities, and buildings between deaths that lay on the linking path. Therefore, if mortality A and B are separated by two buildings, then these structures are also included in the cluster. A linking of deaths had to contain at least 5 mortalities to be classified as a cluster. Again, varying sizes of clusters would have produced different results, but 5 were chosen as the purpose of this exercise was to identify spatial patterns of spread at the street and building level, and larger cluster sizes may have missed many of these macro level associations.

In order to more explicitly identify the impact of cultural distance, clusters identified by the above procedure were classified by dominant subpopulation as long as at least 5 members were drawn from that group. Therefore, for the smallest possible size cluster (containing 5 deaths), all mortalities would have to be of the same nativity for it to be classified as of that nativity.

This cultural connection was extended further by selecting only clusters which had been associated with one subpopulation, and then removing all other mortalities from the cluster. The clustering approach was rerun on the remaining deaths using the previously defined clustering thresholds. Therefore, if a cluster had been identified as "Italian" the new cluster would still have to contain 5 mortalities (all Italian) that could be linked by 50 m in the built environment, and where the mortality sequence was less than 21 days.

Results from this more conservative clustering approach would then be investigated by returning to the GIS and observing the spatial pattern of mortalities and their built environment. Although the numbers involved at this scale would be too small to produce results by any global investigation, it was hoped that visual inspection of the house-to-house disease pattern would reveal further spatial connectivity.

As with all historic epidemiology, one must be careful of misdiagnosis at the time as there was no serological testing. Other sources of error include data entry, cultural reporting biases and epidemic-caused underreporting [27-29] The symptoms of yellow fever and the very fact that this was an epidemic, help raise confidence in the data. Finally, this paper does not attempt to quantify mobility as it would be near impossible to predict pathways through the city with these data.

## Results

The first investigation of the French Quarter involved creating a KD surface to show the overall pattern of disease intensity. This can be seen in Figure 2 where the surface has been contoured, raised 500 m above the three-dimensional rendering of the French Quarter, and then inversely manipulated by multiplying each contour by -0.1. This visualization approach displays high disease concentrations as extending down into the city. The largest inverse peak coincides with an area north east of Jackson Square, the same area that had been revealed by the KD of the entire epidemic, and also by the NNHC for a cluster in August and September (marked by "A" in Figure 1). Although this visualization approach is not commonly applied to disease data, the flexibility of three-dimensions allows the user to explore geographic relationships more fully. For example, the three-dimensional rotational capacity of Arc Scene (ESRI, Redlands, CA) allows this KD cloud to be rotated to view other intensities, some of which also overlapped with the NNHC ellipses for September.

**Figure 3:**
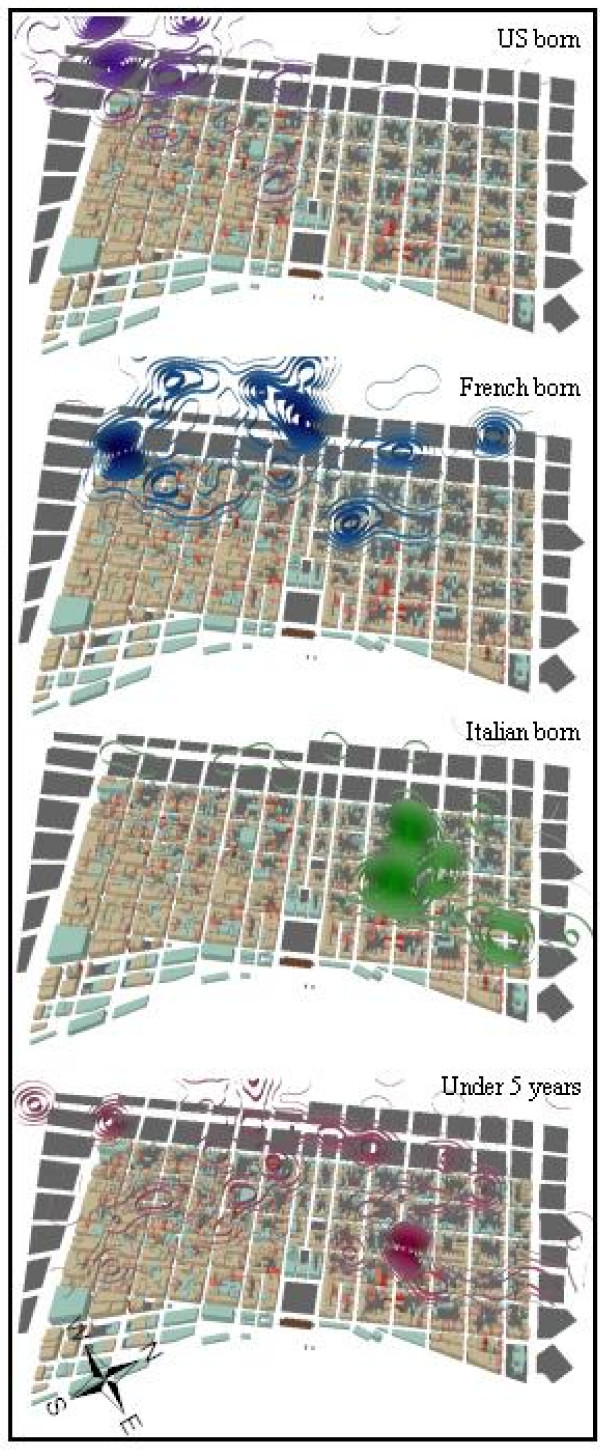
**Kernel density surfaces of US, French, Italian born and children under the age of 5**. The four kernel density surfaces display different geographic patterns of mortality concentration, though the tightest and densest of these occur with Italian deaths. Each of these contoured surfaces has been classified into ten equal categories (allowing for the interpretation that each accounts for approximately 10% of the total disease density), which in turn allows for comparison between the maps (the darker the areas, the more disease), especially in conjunction with Table 1.

Figure 3 displays the KD surfaces for US born, French, Italians and children under the age of 5. Both the KD surfaces for US born and French occupy the area "B" (in Figure 1) which also corresponds to the first NNHC clusters in the French Quarter. The French KD surface also coincides with area "C", and NNHC clusters for September. The Italian KD surface presents the tightest concentration, again coinciding with area "A". The KD surface for children under the age of 5, although having its highest concentration in a similar area as "A", is generally more dispersed across the French Quarter compared to the other three surfaces. From these initial surfaces, it is apparent that the highest concentration of disease across the entire New Orleans epidemic was disproportionately suffered by Italians living in area "A" of the French Quarter. This is supported in Table 1 which compares actual numbers of mortalities and density values for each of the four subgroups.

**Table 1:** Number of mortalities and density by nativity

Nativity or Age	Deaths	KD
USA	77	582
French	99	566
Italian	114	1376
Under 5	108	573

Table 1 displays the total number of deaths falling into each of the four cohorts during the entire epidemic, for just the French Quarter. The table also displays mortality densities (as output from a kernel density surface) for each cohort. The density of Italian mortalities (1376 per square kilometre) far exceeds those of the other three cohorts.

**Figure 4:**
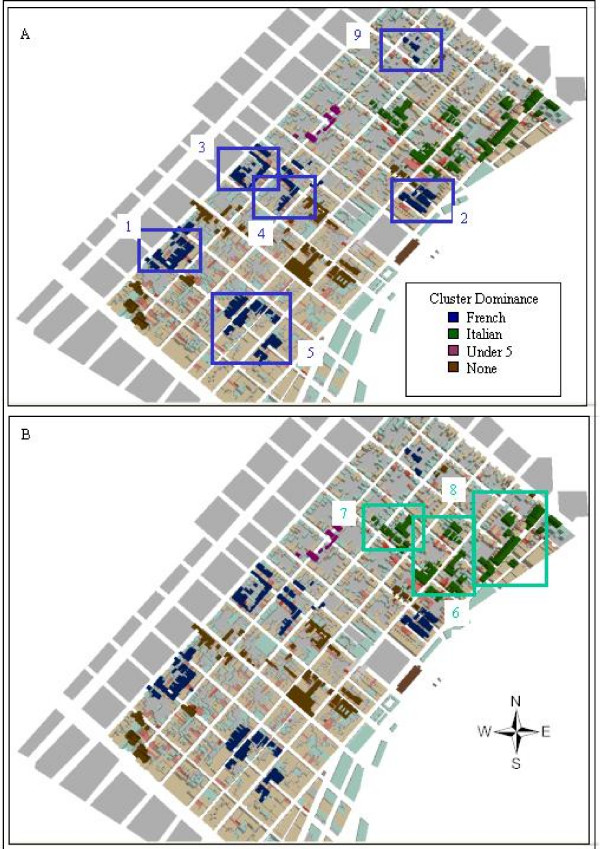
**a and b – Results of a "built-environment" clustering approach with French and Italian nativity members clusters identified**. Twenty-two clusters meeting the combined requirement of deaths being separated by less than 50 m, deaths being less than 21 days apart and groupings having at least 5 members are displayed. These are coloured according to their major cohort, with the six French and three Italian clusters being specifically identified.

Although these KD surfaces show the general spatial pattern of mortalities, they do not capture either temporal complexities or spatial subtlety. In order to investigate this space and time complexity, a cluster approach was developed to incorporate the temporal relationship relevant to yellow fever and the built environment. Using this method, twenty-two clusters (with at least 5 members) were identified, which when mapped by their dominant subgroup revealed a spatio-temporal-cultural sequence. Table 2 reveals the temporal sequence of the cluster groups (the date being the first mortality in the cluster), and Figure 4 shows their location with French and Italian clusters being further identified on the map, with their cluster sequence linked back to Table 2.

**Table 2:** First mortality date and major nativity for each cluster

Dominant nativity	Date of first death	Dominant nativity	Date of first death
French (1)	31^st ^July	Italian (7)*	27^th ^Aug
French (2)	1^st ^Aug	Italian (8)*	29^th ^Aug
None	2^nd ^Aug	None	29^th ^Aug
French (3)	3^rd ^Aug	None	1^st ^Sept
None	13^th ^Aug	None	1^st ^Sept
None	17^th ^Aug	Under 5	2^nd ^Sept
None	18^th ^Aug	None	4^th ^Sept
French (4)	22^nd ^Aug	None	7^th ^Sept
French (5)	22^nd ^Aug	East Europe	7^th ^Sept
None	22^nd ^Aug	None	8^th ^Sept
Italian (6)*	23^rd ^Aug	French (9)	13^th ^Sept

Twenty-two clusters were formed from the mortalities falling in the French Quarter. Of these, six had at least 5 French born members, and three had at least 5 Italian born members. The French clusters grouped together earlier in the epidemic (apart from cluster 9), while the Italian clusters occurred later, though again these were grouped together. Those clusters with an * also remain intact when only single nativity mortalities are considered in their creation (note cluster 6 actually splits into two).

What is interesting from this cluster patterning is that there appears to be disease progression that follows cultural (nativity) connections more so than distance. French cluster 1 and 2 are not geographically proximate, but they do follow sequentially. Similarly, if one were to expect a contagious diffusion, then the Italian Clusters should follow soon after French Cluster 2, but in fact there is a 22 day difference before the first case in Italian Cluster 7. One difference between the French and Italian clusters is that there is a temporal separation between Clusters 1, 2 and 3, and then 4 and 5, while Italian Clusters 6, 7 and 8 are all within 6 days and closely packed together. If we again refer back to the NNHC Figure 1, we can now see that French Cluster 2 contributes to the significant ellipse for August, whereas the later September ellipses overlay well with the Italian Clusters. Indeed, all but one of the other significant ellipses in Figure 1 can be explained by the remaining French Clusters. This final cluster coincides with a Cluster Group that can loosely be tied together by east European nativity.

A further cultural refinement of the clusters methodology only accepts members from the same subpopulation, for example all French or Italian. This results in the original 22 clusters being reduced to six. In addition, one of these original clusters is split into two (Italian Cluster 6) because the distance between the closest elements in either of the sub-clusters is greater than 50 m. Each of these sub clusters still has at least five members. All of the Italian clusters remain, while only two of the six French clusters meet requirements.

**Figure 5:**
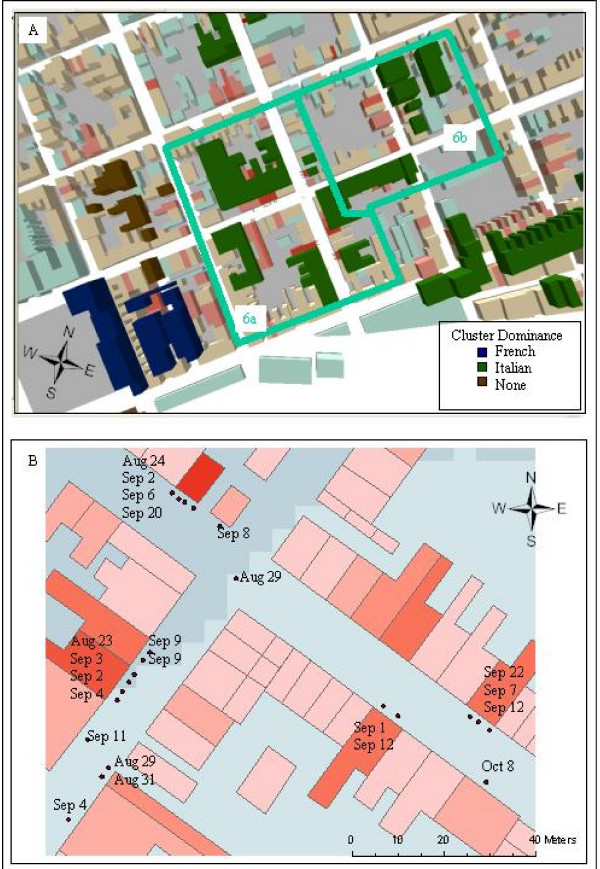
**a and b – A single nativity (Italian) cluster with dates of each mortality**. A more stringent clustering approach only included members from the same nativity while still using the same criteria of deaths being linked by distance and date range. Figure 5a displays one of the resulting clusters which still meets all of the previous cluster requirements, but for only Italian nativity mortalities. This original cluster now forms two sub-clusters (6a and 6b) due to the distance between Italian deaths, and the time frame between these deaths exceeding cluster requirements. Figure 5b considers just the sub cluster 6a, with the dates from all the Italian deaths added. At this scale, three further mortality groupings are evident. The buildings are graded in red according to their number of (all nativity) deaths.

In order to investigate the final (largest) scale of disease patterning, these six clusters were visualized at the street level. Figure 5 shows Italian Cluster 6a (one cluster from former Cluster 6). In addition, the Figure also shows the actual sequence of deaths for this cluster. What can be seen is that at this street-level scale of the cluster, three further sub-groupings can be identified – these all having spatial, temporal, and cultural (nativity) links. The first has 9 mortalities, ranging in date of death from August 23^rd ^to September 11^th^, the second ranging from August 24^th ^to September 8^th^, and the last ranging from September 1^st ^to October 8^th^. This last clustering apparently occurs later in the epidemic sequence than the other two which are more contemporary to each other.

## Discussion

Spatial patterns associated with the yellow fever epidemic of 1878 have been previously discussed in the literature even if not from a spatial analytical perspective. These include the spread from initial point of entry within New Orleans, the spread throughout Louisiana and then along the Mississippi, the spread through neighbourhoods of New Orleans, and concurrently neighbourhoods of Vicksburg, Mississippi, and Memphis Tennessee. Therefore, even though the spatial pattern of an epidemic can be identified and visualized at multiple scales, this final pattern is actually the result of mini-epidemics. Similarly Bailey [30] suggests that most transmissions occur locally with sporadic relocation diffusion.

This paper has shown that the French Quarter emerges as a hotspot in two different citywide analyses. One of these analyses also incorporates the element of time (by month) suggesting that disease was prevalent in this area for a considerable period of the epidemic. A second analysis just considering mortalities within the French Quarter identifies this hotspot to be connected to Italian immigrants. A third analysis designed to create neighbourhood clusters, shed further light on this hotspot by revealing that two nativities were actually involved in the creation of the hotspot; French natives in August, and Italian natives in September. A fourth more stringent cultural clustering approach reveals that one of the mini-epidemics comprising the hotspot can be further subdivided into two sub-clusters, which if visualized on a final building-level map reveals three street-level groups. Therefore, for this one identified hotspot revealed through a city-level analysis, we finally emerge with three neighbouring groups comprised of Italian families.

The clustering approach presented in this paper also reveals further cultural patterns. For the early part of disease progression into the French Quarter, it appears that disease spread through French nativity enclaves. The connection between cases does not follow a typical contagious diffusion processes because two of the first clusters were spatially separated. Instead the spread was connected to nativity. Indeed, the importance of geographic distance only appears to be relevant if neighbourhoods of similar nativity were proximate. Obviously this finding is exploratory and requires more extensive testing, using either other neighbourhoods from the same city and epidemic, or other cities and epidemics as long as data are available in a similar format.

The ability to investigate these scale-dependent patterns to the epidemic, and to consider how the built environment and nativity affect spread, are only made possible by the analysis of historic data within a GIS. This paper has shown that the spatio-cultural living environment of Italian families apparently resulted in an increased disease susceptibility. This finding has not previously received similar attention in commentaries about this epidemic where writers are reliant on contemporary accounts and no subsequent data analysis. What is even more interesting is that in another paper utilizing GIS, Tuckel et al. (2006), describe how southern or eastern European immigrants were more susceptible to the 1918 influenza virus. In their paper they also cite a 1920 study by Winslow and Rogers who specifically identify Italians as being twice as susceptible (in proportion) to the overall epidemic [31]. It would appear, therefore, that this question of ethnic susceptibility should warrant further historic investigation using the advantages of a GIS. Also, as previously mentioned, a lack of denominator data, which in this case includes both morbidity and nativity counts, will limit the interpretation of these results. Further research could use denominator surrogates, either by using city directories, or possibly previous death certificates to create a variation of the proportional mortality ratio [15].

The results of this paper could also provide several research avenues for historians to follow. For example, what was the average length of time since immigration in the cluster neighbourhoods? What were the mobility patterns (daily commutes) and social networks for immigrants living in these cluster areas? To what degree was there neighbourhood segregation based on nativity?

## Conclusion

The relevance of this paper to current epidemiology is in the suggestion of how to protect against a newly emerging infection, or even a bioterrror release. The traditional approach would be a combination of vaccination and cordon sanitaire. The primary rationale for this is space based – a buffer of exclusion being calculated from a distance decay of disease risk. However, this paper suggests (and that of Tuckel et al 2006 who draw similar conclusions) that diffusion occurs based on both cultural distance and geographic distance. Therefore if an outbreak occurs in one ethnic community of Los Angeles, for example, is it more prudent to identify similar cultural areas across the city in which to target intervention as well as simply ring vaccinate? One question for future research would be, *at what geographic distance does this cultural distance finally break down? *In some way this mirrors suggestions by Massad [32] that mass vaccination is less efficacious than targeting known areas with a R_0 _of > 1, which is a basic reproduction number one would expect in neighbourhoods of a high ethnic intensity and therefore social interaction [33].

## Competing interests

The author declares that they have no competing interests.

## Authors' contributions

AC performed all analyses and writing of this paper
